# Clinical presentation and genetic analyses of neurofibromatosis type 1 in independent patients with monoallelic double de novo closely spaced mutations in the *NF1* gene

**DOI:** 10.1002/humu.24423

**Published:** 2022-06-28

**Authors:** Alessandro Stella, Patrizia Lastella, Luigi Viggiano, Rosanna Bagnulo, Nicoletta Resta

**Affiliations:** ^1^ Department of Biomedical Sciences and Human Oncology Laboratory of Medical Genetics, Università di Bari Aldo Moro Bari Italy; ^2^ Rare Disease Center Internal MedicineUnit ‘C. Frugoni’, AOU Policlinico di Bari Bari Italy; ^3^ Department of Biology University of Bari Aldo Moro Bari Italy

**Keywords:** closely spaced multiple mutations, de novo mutations, *in cis* doublets, neurofibromatosis type 1, paternal age effect

## Abstract

Neurofibromatosis type 1 (NF1) belongs to RASopathies, a group of syndromes caused by germline mutations in Ras/MAPK pathway genes. Most NF1 patients exhibit single inactivating pathogenic variants within the *NF1* gene. We performed extensive genetic analyses in two NF1 families disclosing the first two cases of double de novo monoallelic *NF1* variants. Both index patients described in this study had classical NF1. Probands were born from fathers in their late 30s and presented closely spaced double mutations (<100 bp) in *NF1* regions showing an excess of somatic mutations. Closely spaced multiple mutations have been reported in RAS/MAPK signaling genes but never in *NF1*. Mutagenesis is a quasi‐random process in humans, therefore two causative variants in the same gene, moreover in the same allele are exceptional. Here, we discuss possible mechanisms for this ultrarare event. Our findings confirm the possibility of a higher risk of concurrent de novo variants in *NF1*.

Neurofibromatosis type 1 (NF1 [OMIM: 162200]) is one of the most common genetic diseases with an estimated prevalence of 1 in 3000 (Friedman, 1993). Inherited as autosomal dominant, it is caused by mutations in the large *NF1* gene spanning 58 exons encoding for neurofibromin, a 2839 amino acids protein. NF1 is clinically diagnosed in accord with the recently revised diagnostic criteria (Legius et al., 2021).

In 50% of cases, *NF1* mutations arise de novo, and diagnosis is frequently suspected in prepuberal years, thus a correct variant interpretation is crucial for a reliable NF1 assessment. In diagnostic laboratories, variant pathogenicity is assessed in accord with the American College of Medical Genetics and Genomics (ACMG) and the Association for Molecular Pathology (AMP) (Richards et al., 2015). *NF1* pathogenic variants (PVs) can occur across the entire coding region and include virtually the full range of various types from multiexons deletion/duplication, including whole gene deletions, to missense mutations (Messiaen, 2020). In addition, most missense variants are still classified as variants of uncertain clinical significance (VUS), and little has been reported on the correlation between variants’ position and their pathogenic effects (Accetturo et al., 2020; Koczkowska et al., 2020, 2018).

A second *NF1* hit, and consequent loss‐of‐function, has been documented in several *NF1*‐associated cancers (Boudry‐Labis et al., 2013; Uusitalo et al., 2016), in cutaneous and plexiform neurofibromas as well as in tissues from pigmentary nonneoplastic lesions such as cafè‐au‐lait macules (CALMs) (De Schepper et al., 2008). A phenotype mimicking NF1 is caused by homozygous mutations in mismatch repair genes in a novel clinical entity known as constitutional mismatch repair deficiency (Alotaibi et al., 2008; Raevaara et al., 2004; Urganci et al., 2015).

Here, we present the first two independent cases of NF1 patients with de novo in *cis* double mutations in the *NF1* gene with a classical NF1 phenotype.

The first patient was a male individual in his 50s, displaying several cutaneous neurofibromas, bilateral axillary freckling, and no CALMs. Therefore, he was diagnosed with NF1 in accord with National Institutes of Health guidelines. Four years before counseling the patient had a surgically removed gastrointestinal stromal tumor (GIST) in the jejunum and gastric fundus. No adjuvant therapy was recommended after surgery, and the patient has been undergoing periodic follow‐ups since. The patient's parents had no history of NF1 and were deceased in their 90s of natural causes. At the patient's birth, the father was 38 years old while the mother was 32 years old. After genetic counseling, a next‐generation sequencing (NGS) multigene panel was used (*A2ML1, BRAF, CBL, HRAS, KRAS, MAP2K1, MAP2K2, NF1, NRAS, PTPN11, RAF1, RIT1, SHOC2, SOS1*, and *SPRED1*) to search for the presence of causative mutations in RASopathies associated genes.

The second case was a 6 years old girl who was referred to our unit with >20 CALMs and bilateral inguinal and axillary freckling (Supporting Information: Figure S1a–d). In addition, slight diffuse hypertrichosis was present (Supporting Information: S1e,f). Both parents did not show any clinical features suggestive of NF1. The paternal age at the daughter's birth was again 38 years old. while the mother was in her late 20s. After genetic counseling NGS gene testing was performed with the same multi‐gene panel mentioned previously.

In Case 1, the NGS analysis disclosed two PVs in the *NF1* gene (reference sequence: NM_000267.3): a c.3198‐1G>A splicing mutation (genomic coordinates in GRCh38/hg38: chr 17: 31232072G>A) and a c.3295A>T p.(lys1099*) nonsense mutation in the 3’‐adjacent exon 25 (genomic coordinates in GRCh38/hg38: chr 17: 31232170A>T). Both variants were confirmed with Sanger sequencing (Figure 1a). After gene testing, the patient's only daughter, in her late 20s, was invited to a genetic counseling session during which several CALMs >1.5 cm, diffuse cutaneous neurofibromas, bilateral axillary freckling, and a plexiform neurofibroma on her left calf were detected. Sanger sequencing of *NF1* exon 25 confirmed the presence of both variants in the patient's daughter suggesting they were *in cis*. The proband's DNA was amplified with primers flanking the two variants and the PCR product was cloned into the TA vector. Sequencing of 20 recombinant clones had invariably either the normal sequence or both variants confirming that they were *in cis* on the same allele (Figure 1b). The c.3198‐1G>A nucleotide change is predicted to disrupt the splicing acceptor (SA) site according to all predictors in the Alamut® Splicing module (Figure 1c). To confirm the variant's effect on splicing the complementaryDNA obtained from the patient's and his daughter's blood samples was sequenced and, in both, revealed an aberrant mRNA containing the r.3198_3199delAG mutation predicted to cause a p.(Asp1067Phefs*21) at protein level (Figure 1d). Therefore, the c.3198‐1G>A mutation cannot be considered a compensatory mutation, since the novel splicing site created offsets the normal open reading frame resulting in a similarly truncated NF1 protein.

**Figure 1:**
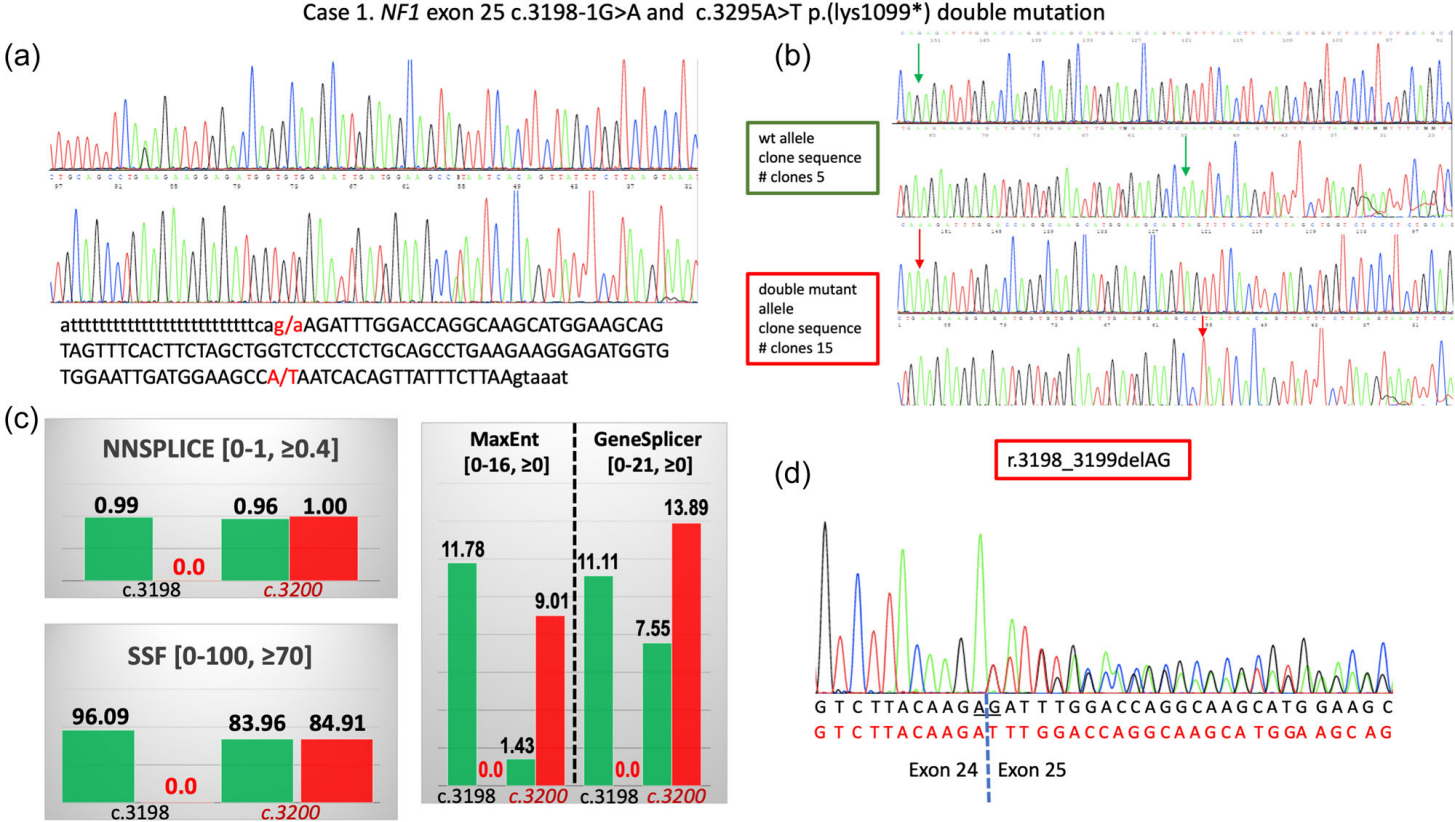
Molecular analyses on Case 1 de novodouble mutation. (a) Electropherogram showing the *NF1* variants c.3198‐1G>A and c.3295A>T p.(lys1099*) identified with NGS and confirmed with Sanger sequencing. Below the electropherograms is indicated the exon 25 sequence. Nucleotide changes are indicated with arrows in the electropherogram and in red in the exon sequence. (b) sequencing results of the cloning of PCR products from a patient's DNA. Green arrows point at wild‐type nucleotides, red arrows at mutated nucleotides. The number of clones sequenced with either wild‐type or mutated sequence is indicated. (c) Alamut® Visual Splicing predictions for variant c.3198‐1G>A, transcript NM_000267.3. The analysis range is from c.3198‐106 (intron24) to c.3303 (exon 25). Green bars represent scores for the normal sequence, and red bars score for the mutated sequence. c.1398 is the position of the first de novo mutation in exon 25, c.3200 is the position of a novel splice site created as a consequence of mutation. In square brackets, the range for scores of splicing predictors, and the threshold for a significant effect on splicing. (d) electropherogram of Sanger sequencing on patient's 2 mRNA purified from peripheral blood collected in PAXgene® blood RNA tubes (PreAnalytiX GmbH). In black, the sequence of the normal cDNA with deleted nucleotides is underlined; in red, the mutated cDNA sequence. The mutated cDNA is generated by the usage of the novel splice site created at the genomic level. cDNA, complementary DNA; NGS, next‐generation sequencing.

In Case 2, NGS disclosed the presence of two *NF1* variants in exon 21: a frameshifting mutation c.2546del p.(Gly849GlufsTer29) (genomic coordinates in GRCh38/hg38: chr 17: 31229161del), and the missense variant c.2548G>A p.(Val850Met) (genomic coordinates in GRCh38/hg38: chr 17: 31229163G>A). This second missense variant has never been reported before and is classified as likely pathogenic by the Varsome variant impact assessment tool (accessed November 4th, 2021), which implements the ACMG/AMP rules. Specifically, the Val850Met lies in a region of 17 amino‐acids length which has 14 non‐VUS missense/in‐frame variants (14 pathogenic and 0 benign, ACMG/AMP rule PM1), and therefore is considered a mutation hotspot. Furthermore, it is not present in gnomAD (ACMG_AMP rule PM2), and is predicted to be pathogenic by 9 of 12 variant predictions (ACMG/AMP rule PP3). Also, the c.2548G>A variant had scores equal to or above the cutoff values for pathogenicity, as previously defined (Accetturo et al., 2020), for 5/5 of cutoff performance indicators (ClinPred), and for 3/5 (REVEL and VEST4).

The two variants were again confirmed with Sanger sequencing (Figure 2a). Since the first of these two variants is located in a run of five consecutive guanines, the exact position of this nucleotide change cannot be precisely determined and may lie anywhere from −2 to −7 with respect to the subsequent missense variant. Sequencing analysis on the girl's parent DNA, and an allele‐specific PCR confirmed the presence of the two variants only in the proband (Figure 2b). Finally, as previously described, the girl's DNA was amplified with primers flanking the exon and the PCR product cloned in the TA vector. The sequencing of 20 recombinant clones had again invariably either wild‐type sequence or both variants (Figure 2c).

**Figure 2:**
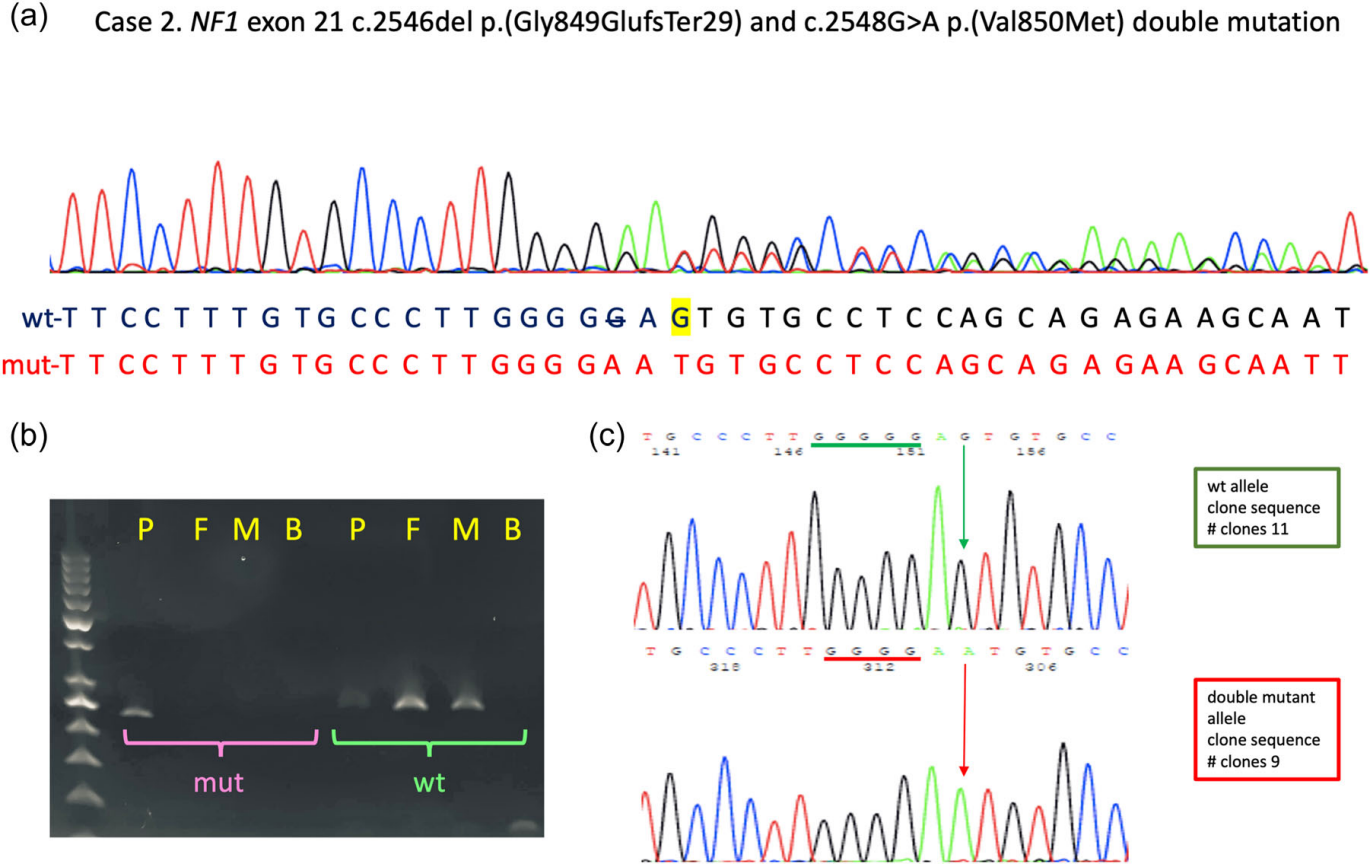
Molecular analyses on Case 2 de novo double mutation. (a) Electropherogram showing the *NF1* variants c.2546del p.(Gly849GlufsTer29) and c.2548G>A p.(Val850Met) identified with NGS and confirmed with Sanger sequencing; (b) Allele‐specific PCR on proband's DNA (P), her father (F), her mother (M) and blank control (B). mut is the amplification with mutation‐specific primer and wt is the amplification with wild‐type‐specific primer; (c) sequencing results of the cloning of PCR products from patient's DNA. The five consecutive guanines in the wt sequence and the four in the c.2546del mutations are highlighted by green and red lines, respectively. The wt and mut nucleotides for mutation c.2548G>A are indicated by green and red arrows, respectively. The number of clones sequenced with either wt or mut sequence is indicated. NGS, next‐generation sequencing.

Next, we inspected the sequence surrounding the two double variants with nonB‐DB (https://nonb-abcc.ncifcrf.gov/apps/site/default), since it has been reported that non‐B DNA structures could favor the generation of multiple mutations (Chen et al., 2009). However, no non‐B DNA structures were identified in both regions. Then, we used the UNAFold web server (http://www.unafold.org/) to verify whether the two regions might assemble secondary structures. UNAFold returned one structure for exon 21 (Supporting Information: Figure S2) and five for exon 25 (Supporting Information: Figure S3a–e). In all structures, variants were close to stem‐loop switch regions. Interestingly, in one of the five structures built by UNAFold for exon 25, the two variants spaced 98 nucleotides apart were in close proximity (Supporting Information: Figure S3e).

To our knowledge, the two patients described here are the first reported carriers of double de novo mutations in the same *NF1* allele. The three patients presented in this study had a rather classical NF1 phenotype. However, in Case 1, a GIST was previously diagnosed. GIST has been occasionally reported in NF1 and is present in 6%–8% of NF1 patients (Vargas Ávila et al., 2021). The patient's daughter was diagnosed with NF1 at the time of the genetic counseling session since she presented typical NF1 clinical signs. In Case 2, the presence of cutaneous symptoms only (CALMs and axillary freckling) is in line with the age‐dependent penetrance of more easily recognizable NF1 stigmata such as cutaneous or plexiform neurofibromas. As previously mentioned, the girl also had mild diffuse hirsutism which has been sporadically reported in NF1, and mostly colocalizing with cutaneous neurofibromas (Friedrich & Hagel, 2018; Ortonne et al., 2018; Praxedes et al., 2010).

Interestingly, despite the large coding sequence of the *NF1* gene, in both cases, the concurrent *in cis* variants were closely spaced (98 nucleotides in Case 1 and 2–7 nucleotides in Case 2) and in the same exon (exons 21 and 25, respectively). Sequence inspection did not reveal a clear mutational hotspot excluding the long poly‐T stretch (28 nt) immediately preceding exon 25. However, the two de novo variants identified in exon 25 were both single nucleotide substitutions rather than indel changes as expected if variants were the result of DNA polymerase slippage errors caused by the poly‐T sequence.

The presence of multiple mutations in close proximity has been found in several human genes and dubbed as closely spaced multiple mutations (Chen et al., 2009). In particular, pair of mutations separated by less than 100 bp, as in our two cases, have been hypothesized to represent a signature of transient hypermutability in human genes. While no obvious mutation hotspots have been identified in *NF1*, in COSMIC (https://cancer.sanger.ac.uk/cosmic/gene/analysis?ln=NF1#distribution), accessed on January 13th, 2022) there were 22 nucleotide substitutions targeting the SA site of exon 25 (intronic position −1 to −3), of which three were c.3198‐1G>A. In contrast, only three substitutions were listed in ClinVar at exon 25 SA (c.3198‐1G>A not present, assessed on September 13th). We focused on the last three positions of the preceding intron since these positions are significantly associated with splicing deregulation (Stella et al., 2018). Similarly, the exon 21 c.2546del, is present twice in COSMIC, and together with the c.2548G>A lies in a stretch of 21 coding nucleotides hosting 26 mutations in COSMIC (*NF1* coding sequence from c.2525 to c.2546). This would result in a local mutation ratio of 1.24 mutations/nucleotide compared to 0.39 mutations/nucleotide for the remaining 417 nucleotides of exon 21. In this 21‐nucleotide sequence, there are contiguous GG, TT (2), CC, TTT, CCC, and the final GGGGG repeats that may render the region particularly prone to replication or misalignment errors. Two de novo mutations occur in two different NF1 families with two *NF1* PVs lying 80 and 3 nucleotides, respectively, upstream of our doublet de novo c.2546del, c.2548G>A have been recently reported (Garcia et al., 2022) (Supporting Information: Figure S4). This finding might confirm that the region could be frequently targeted by de novo *NF1* variants.

Thus, it is possible that the two variants were the outcome of a simultaneous or a quasi‐simultaneous mutational event in regions prone to replication errors. Considering that the closest of the five consecutive guanines lies two nucleotides upstream of the following c.2548G>A, the possibility of single mutational events generating a complex allele cannot be ruled out. It should be added that, differently from exon 25 splicing variants, 20 variants in the same 21 nucleotide stretch were also present in ClinVar.

Recently, combining ultradeep sequencing with a low‐frequency variant prioritization strategy, 61 different variants were identified in the testes of five aged men (Maher et al., 2018). Eighty percent of variants were found in genes encoding for a protein involved in the RAS/MAPK pathway. Furthermore, a clonal expansion of already mutated male reproductive precursor cells, i.e. spermatogonia, has been described in achondroplasia, Apert, Noonan, and Costello syndrome (Goriely & Wilkie, 2012). This phenomenon has been named “selfish spermatogonial selection” and is associated with high rates of de novo mutations and strong paternal age effects (PAE). A further feature of selfish selection is the frequent occurrence of multiple nucleotide substitutions that have been documented in most PAE disorders (reviewed in Goriely & Wilkie, 2012). NF1 shares overlapping symptoms with Costello and Noonan syndromes, and similarly encodes for a key regulator of the RAS/MAPK pathway. In NF1, as in other RASopathies, although yet debated, increasing paternal age has been associated with a higher risk for de novo mutations (Adel Fahmideh et al., 2018; Dubov et al., 2016). Interestingly, in both our cases, fathers were 38 years old at probands’ birth, therefore at the first peak of the curve correlating the incidence of new mutations in *NF1* and parental age in one of the first studies analyzing this correlation (Risch et al., 1987). In the abovementioned report, both Apert syndrome and neurofibromatosis showed an increase up to age 37, a sudden drop at age 42, and a new increase at age 47. Recently, the co‐occurrence of two independent biallelic *NF1* changes has been documented in five NF1 families (Pacot et al., 2019). Also, a very recent work reports four different families where two different NF1 disease‐causing variants were detected. In all families, a de novo mutation was present in the offspring of an affected NF1 male, and in two, the paternal origin of the de novo mutation could be demonstrated (Garcia et al., 2022). However, multiple de novo mutations occurring in the same *NF1* allele have never been described. Differently from all known PAE disorders which are caused by activating mutations, *NF1* acts as a classical tumor suppressor gene with most PVs causing a low protein abundance (Long et al., 2022). Thus, different lines of evidence point to a crucial role of RAS/MAPK/ERK pathway dysregulation in spermatogonial stem cells homeostasis. Being part of the RAS signaling pathway, implicated in oncogenesis, and highly expressed in the testis (https://gtexportal.org/home/gene/NF1) could target *NF1* for having a role in this phenomenon.

It has to be said that the double de novo *NF1* variants we report, being on the same allele, are unlikely to confer an additive growth advantage in spermatogonial cells. Rather, they could represent the result of serendipitous events in a gene locus with a high mutation rate (Garcia et al., 2022).

Three of the *NF1* variants identified in our two families are novel while the c.2546del has been already reported. None of the four variants was present in GnomAD (last accessed on October 30th, 2021). The c.3198‐1G>A, the nonsense c.3295A>T, and the c.2546del are all clearly pathogenic whereas, as discussed before, several lines of evidence show that also the c.2548G>A could be pathogenic.

The two double de novo variants are both localized in regions of the *NF1* gene which appear to represent a mutation hotspots in oncogenesis, a process somehow recapitulated by the selfish selection in the testis. However, as mentioned previously, the concurrent double changes could also be the result of a single event targeting regions prone to replication errors.

Finally, the possibility of a first *NF1* mutation followed by a quick second mutational event in the same cell clone has been proposed to explain isolated segmental monoclonal two‐hit mosaicism in NF1 (Torrelo & Happle, 2021).

Our study has the following limitations. First, given the available biological samples, we cannot formally rule out that in one or both cases the double *in cis*, de novo variants could be originated in an early post‐zygotic stage. Second, we could not unequivocally determine the parental origin of the mutations even though this topic was analyzed by several experimental means (see Supporting Information Text). Lastly, considering the type of variants we have identified, we cannot formally ascribe a specific underlying mechanism(s) of mutagenesis. In fact, the four variants reported are not homocoordinate mutations (i.e., multiple mutations of the same type occurring *in cis* at different sites) for which different mechanisms of mutagenesis have been hypothesized (Chen et al., 2009).

These findings could have important consequences in NF1 genetic counseling considering the possibility of an increased risk for de nov*o* mutations associated with paternal age. The de novo doublets reported here, and the recently described four NF1 families with two PVs (two of them paternal in origin), should also alert to the possibility of failure in detecting NF1 causative variants once the presence of parentally derived ones has been excluded (Garcia et al., 2022). While the *NF1* doublets we identified, since *in cis*, have not influenced the clinical phenotype of our patients, it is yet possible that in other genes the same type of situation could explain apparent genotype‐phenotype incongruities.

Finally, the two independent cases of double de novo variants in the *NF1* gene could lend support to the important role of RAS/MAPK signaling in the spermatogonial homeostasis and the predatory clonal expansion of cells carrying selfish PVs.

## CONFLICT OF INTEREST

The authors declare no conflict of interest.

## WEB RESOURCES

LOVD *NF1* database: https://databases.lovd.nl/shared/genes/NF1; ClinVar *NF1* database: https://www.ncbi.nlm.nih.gov/clinvar/?term=NF1 [gene]; Varsome *NF1* database: https://varsome.com/gene/hg38/NF1; Non‐B structures database (nonB‐DB): https://nonb-abcc.ncifcrf.gov/apps/site/default; UNAFold web server for secondary structures assembly: http://www.unafold.org/; COSMIC *NF1* database: https://cancer.sanger.ac.uk/cosmic/gene/analysis?ln=NF1#distribution; GTEX database for *NF1* expression: https://gtexportal.org/home/gene/NF1; GnomAD: https://gnomad.broadinstitute.org/gene/ENSG00000196712?dataset=gnomad_r2_1

## Supporting information

Supporting information.

## References

[humu24423-bib-0001] Accetturo, M. , Bartolomeo, N. , & Stella, A. (2020). In‐silico analysis of *NF1* missense variants in ClinVar: Translating variant predictions into variant interpretation and classification. International Journal of Molecular Sciences, 21(3), 721. 10.3390/ijms21030721 PMC7037781 31979111

[humu24423-bib-0002] Adel Fahmideh, M. , Tettamanti, G. , Lavebratt, C. , Talbäck, M. , Mathiesen, T. , Lannering, B. , Johnson, K. J. , & Feychting, M. (2018). Parental age and risk of genetic syndromes predisposing to nervous system tumors: Nested case‐control study. Clinical Epidemiology, 10, 729–738. 10.2147/CLEP.S159183 29950902 PMC6016487

[humu24423-bib-0003] Alotaibi, H. , Ricciardone, M. D. , & Ozturk, M. (2008). Homozygosity at variant MLH1 can lead to secondary mutation in NF1, neurofibromatosis type I and early onset leukemia. Mutation Research/DNA Repair, 637(1–2), 209–214. 10.1016/j.mrfmmm.2007.08.003 17889038

[humu24423-bib-0004] Boudry‐Labis, E. , Roche‐Lestienne, C. , Nibourel, O. , Boissel, N. , Terre, C. , Perot, C. , Eclache, V. , Gachard, N. , Tigaud, I. , Plessis, G. , Cuccuini, W. , Geffroy, S. , Villenet, C. , Figeac, M. , Leprêtre, F. , Renneville, A. , Cheok, M. , Soulier, J. , Dombret, H. , & Preudhomme, C. , French ALFA Group . (2013). Neurofibromatosis‐1 gene deletions and mutations in de novo adult acute myeloid leukemia. American Journal of Hematology, 88(4), 306–311. 10.1002/ajh.23403 23460398

[humu24423-bib-0005] Chen, J. M. , Férec, C. , & Cooper, D. N. (2009). Closely spaced multiple mutations as potential signatures of transient hypermutability in human genes. Human Mutation, 30(10), 1435–1448. 10.1002/humu.21088 19685533

[humu24423-bib-0006] De Schepper, S. , Maertens, O. , Callens, T. , Naeyaert, J. M. , Lambert, J. , & Messiaen, L. (2008). Somatic mutation analysis in NF1 cafe au lait spots reveals two NF1 hits in the melanocytes. Journal of Investigative Dermatology, 128(4), 1050–1053. 10.1038/sj.jid.5701095 17914445

[humu24423-bib-0007] Dubov, T. , Toledano‐Alhadef, H. , Bokstein, F. , Constantini, S. , & Ben‐Shachar, S. (2016). The effect of parental age on the presence of de novo mutations ‐ Lessons from neurofibromatosis type I. Molecular Genetics & Genomic Medicine, 4(4), 480–486. 10.1002/mgg3.222 27468422 PMC4947867

[humu24423-bib-0008] Friedman, J. M. (1993). Neurofibromatosis 1. In M. P. Adam , H. H. Ardinger , R. A. Pagon , S. E. Wallace , L. J. H. Bean , K. Stephens , & A. Amemiya (Eds.), GeneReviews®. University of Washington.

[humu24423-bib-0009] Friedrich, R. E. , & Hagel, C. (2018). Pigmented (melanotic) diffuse neurofibroma of the back in neurofibromatosis type 1. GMS Interdisciplinary Plastic and Reconstructive Surgery DGPW, 7, Doc04. 10.3205/iprs000124 30112270 PMC6073164

[humu24423-bib-0010] Garcia, B. , Catasus, N. , Ros, A. , Rosas, I. , Negro, A. , Guerrero‐Murillo, M. , Valero, A. M. , Duat‐Rodriguez, A. , Becerra, J. L. , Bonache, S. , Lázaro Garcia, C. , Comas, C. , Bielsa, I. , Serra, E. , Hernández‐Chico, C. , Martin, Y. , Castellanos, E. , & Blanco, I. (2022). Neurofibromatosis type 1 families with first‐degree relatives harbouring distinct *NF1* pathogenic variants. Genetic counselling and familial diagnosis: What should be offered? *Journal of Medical Genetics* Advance online publication. 10.1136/jmedgenet-2021-108301 35121649

[humu24423-bib-0011] Goriely, A. , & Wilkie, A. O. (2012). Paternal age effect mutations and selfish spermatogonial selection: Causes and consequences for human disease. American Journal of Human Genetics, 90(2), 175–200. 10.1016/j.ajhg.2011.12.017 22325359 PMC3276674

[humu24423-bib-0012] Koczkowska, M. , Callens, T. , Chen, Y. , Gomes, A. , Hicks, A. D. , Sharp, A. , Johns, E. , Uhas, K. A. , Armstrong, L. , Bosanko, K. A. , Babovic‐Vuksanovic, D. , Baker, L. , Basel, D. G. , Bengala, M. , Bennett, J. T. , Chambers, C. , Clarkson, L. K. , Clementi, M. , Cortés, F. M. , … Messiaen, L. M. (2020). Clinical spectrum of individuals with pathogenic NF1 missense variants affecting p.Met1149, p.Arg1276, and p.Lys1423: Genotype‐phenotype study in neurofibromatosis type 1. Human Mutation, 41(1), 299–315. 10.1002/humu.23929 31595648 PMC6973139

[humu24423-bib-0013] Koczkowska, M. , Chen, Y. , Callens, T. , Gomes, A. , Sharp, A. , Johnson, S. , Hsiao, M. C. , Chen, Z. , Balasubramanian, M. , Barnett, C. P. , Becker, T. A. , Ben‐Shachar, S. , Bertola, D. R. , Blakeley, J. O. , Burkitt‐Wright, E. M. M. , Callaway, A. , Crenshaw, M. , Cunha, K. S. , Cunningham, M. , … Messiaen, L. M. (2018). Genotype‐phenotype correlation in NF1: Evidence for a more severe phenotype associated with missense mutations affecting NF1 codons 844‐848. American Journal of Human Genetics, 102(1), 69–87. 10.1016/j.ajhg.2017.12.001 29290338 PMC5777934

[humu24423-bib-0014] Legius, E. , Messiaen, L. , Wolkenstein, P. , Pancza, P. , Avery, R. A. , Berman, Y. , Blakeley, J. , Babovic‐Vuksanovic, D. , Cunha, K. S. , Ferner, R. , Fisher, M. J. , Friedman, J. M. , Gutmann, D. H. , Kehrer‐Sawatzki, H. , Korf, B. R. , Mautner, V. F. , Peltonen, S. , Rauen, K. A. , Riccardi, V. , Yohay, K. , Huson, S. M. , Evans, D. G. , … Plotkin, S. R. (2021). Revised diagnostic criteria for neurofibromatosis type 1 and Legius syndrome: An international consensus recommendation. Genetics in Medicine, 23(8), 1506–1513. 10.1038/s41436-021-01170-5 34012067 PMC8354850

[humu24423-bib-0015] Long, A. , Liu, H. , Liu, J. , Daniel, M. , Bedwell, D. M. , Korf, B. , Kesterson, R. A. , & Wallis, D. (2022). Analysis of patient‐specific NF1 variants leads to functional insights for Ras signaling that can impact personalized medicine. Human Mutation, 43(1), 30–41. 10.1002/humu.24290 34694046

[humu24423-bib-0016] Maher, G. J. , Ralph, H. K. , Ding, Z. , Koelling, N. , Mlcochova, H. , Giannoulatou, E. , Dhami, P. , Paul, D. S. , Stricker, S. H. , Beck, S. , McVean, G. , Wilkie, A. O. M. , & Goriely, A. (2018). Selfish mutations dysregulating RAS‐MAPK signaling are pervasive in aged human testes. Genome Research, 28(12), 1779–1790. 10.1101/gr.239186.118 30355600 PMC6280762

[humu24423-bib-0017] Messiaen, L. (2020In: Molecular diagnosis of NF1. In G. Tadini , E. Legius , & H. Brems (Eds.), Multidisciplinary approach to neurofibromatosis 1. Springer.

[humu24423-bib-0018] Ortonne, N. , Wolkenstein, P. , Blakeley, J. O. , Korf, B. , Plotkin, S. R. , Riccardi, V. M. , Miller, D. C. , Huson, S. , Peltonen, J. , Rosenberg, A. , Carroll, S. L. , Verma, S. K. , Mautner, V. , Upadhyaya, M. , & Stemmer‐Rachamimov, A. (2018). Cutaneous neurofibromas: Current clinical and pathologic issues. Neurology, 91(2 Suppl 1), S5–S13. 10.1212/WNL.0000000000005792 29987130

[humu24423-bib-0019] Pacot, L. , Burin des Roziers, C. , Laurendeau, I. E. , Briand‐Suleau, A. , Coustier, A. , Mayard, T. , Tlemsani, C. , Faivre, L. , Thomas, Q. , Rodriguez, D. , Blesson, S. , Dollfus, H. , Muller, Y. G. , Parfait, B. , Vidaud, M. , Gilbert‐Dussardier, B. , Yardin, C. , Dauriat, B. , Derancourt, C. ,, … Pasmant, E. (2019). One *NF1* mutation may conceal another, Genes, 10(9), 633. 10.3390/genes10090633 PMC6769760 31443423

[humu24423-bib-0020] Praxedes, L. A. , Pereira, F. M. , Mazzeu, J. F. , Costa, S. S. , Bertola, D. R. , Kim, C. A. , Vianna‐Morgante, A. M. , & Otto, P. A. (2010). An illustrative case of neurofibromatosis type 1 and NF1 microdeletion. Molecular Syndromology, 1(3), 133–135. 10.1159/000319976 21031083 PMC2957849

[humu24423-bib-0021] Raevaara, T. E. , Gerdes, A. M. , Lönnqvist, K. E. , Tybjaerg‐Hansen, A. , Abdel‐Rahman, W. M. , Kariola, R. , Peltomäki, P. , & Nyström‐Lahti, M. (2004). HNPCC mutation MLH1 P648S makes the functional protein unstable, and homozygosity predisposes to mild neurofibromatosis type 1. Genes, Chromosomes & Cancer, 40(3), 261–265. 10.1002/gcc.20040 15139004

[humu24423-bib-0022] Richards, S. , Aziz, N. , Bale, S. , Bick, D. , Das, S. , Gastier‐Foster, J. , Grody, W. W. , Hegde, M. , Lyon, E. , Spector, E. , Voelkerding, K. , & Rehm, H. L. , ACMG Laboratory Quality Assurance Committee . (2015). Standards and guidelines for the interpretation of sequence variants: A joint consensus recommendation of the American College of Medical Genetics and Genomics and the Association for Molecular Pathology. Genetics in Medicine, 17(5), 405–424. 10.1038/gim.2015.30 25741868 PMC4544753

[humu24423-bib-0023] Risch, N. , Reich, E. W. , Wishnick, M. M. , & McCarthy, J. G. (1987). Spontaneous mutation and parental age in humans. American Journal of Human Genetics, 41(2), 218–248. 3618593 PMC1684215

[humu24423-bib-0024] Stella, A. , Lastella, P. , Loconte, D. C. , Bukvic, N. , Varvara, D. , Patruno, M. , Bagnulo, R. , Lovaglio, R. , Bartolomeo, N. , Serio, G. , & Resta, N. (2018). Accurate classification of *NF1* gene variants in 84 Italian patients with neurofibromatosis type 1, Genes, 9(4), 216. 10.3390/genes9040216 PMC5924558 29673180

[humu24423-bib-0025] Torrelo, A. , & Happle, R. (2021). A proposed new category of cutaneous segmental mosaicism: Isolated segmental biallelic monoclonal mosaicism. Journal of the European Academy of Dermatology and Venereology, 35(4), e265–e267. 10.1111/jdv.17008 33078473

[humu24423-bib-0026] Urganci, N. , Genc, D. B. , Kose, G. , Onal, Z. , & Vidin, O. O. (2015). Colorectal cancer due to constitutional mismatch repair deficiency mimicking neurofibromatosis I. Pediatrics, 136(4), e1047–e1050. 10.1542/peds.2015-1426 26391938

[humu24423-bib-0027] Uusitalo, E. , Rantanen, M. , Kallionpää, R. A. , Pöyhönen, M. , Leppävirta, J. , Ylä‐Outinen, H. , Riccardi, V. M. , Pukkala, E. , Pitkäniemi, J. , Peltonen, S. , & Peltonen, J. (2016). Distinctive cancer associations in patients with neurofibromatosis type 1. Journal of Clinical Oncology, 34(17), 1978–1986. 10.1200/JCO.2015.65.3576 26926675

[humu24423-bib-0028] Vargas Ávila, A. L. , Jiménez Leyva, A. , Vargas Flores, J. , Reyes Garcia, V. G. , de Alba Cruz, I. , Narváez González, H. F. , & Galicia Gómez, T. J. (2021). GIST associated with von recklinghausen disease: Report of two cases and review of literature. Annals of Medicine and Surgery, 62, 365–368. 10.1016/j.amsu.2021.01.033 33552495 PMC7848713

