# Supplementary material for: Clinical presentation and genetic analyses of neurofibromatosis type 1 in independent patients with monoallelic double de novo closely spaced mutations in the NF1 gene
Source: Hum Mutat. 2022 Jun 28;43(10):1354–60. doi: 10.1002/humu.24423 (PMC9540858; doi:10.1002/humu.24423)
Supplement: Supplementary file 1 — Supporting information. [file HUMU-43-1354-s001.pdf]

SUPPLEMENTARY TEXT

We explored all the possibilities to identify the parent-of-origin of the double mutations described. First, we carefully inspected the VCF/BAM files from family 2 trio and we noticed that unfortunately all SNPs included in our NGS multigene panel were not informative. Therefore, we expanded our strategy to detect the parent-of-origin for family 2 trio double mutations by using Unfazed (Belyeu et al., 2021) on the targeted gene sequencing raw data (VCF, BAM files) from the trio. However, all SNPs in the targeted NF1 region were again uninformative. Next, we sought to use the two SNPs rs8067021 and rs2071009 which were used by Garcia et al., and which were not included in our NGS target region, to verify if we could determine the mutations' parent-of-origin. Again, not unsurprisingly, these two SNPs were uninformative in our trio.

Finally, we carefully checked the work from Goldmann et al. (Goldman et al., 2018). Here are specifically described clustered de novo mutations (cDNMs) that are different from closely spaced multiple mutations (CSMMs) such as those we report. As noted by Goldmann et al, it is evident that fathers are clearly the parent-of-origin for DNMs with intermutational distance up to 50bp. A striking "maternal" origin effect is evident only for DNMs spaced 1-5kb and to a lesser degree for DNMs distant 5-20kb (as paper authors comments at page 488). Further, authors detail the pattern of cDNMs across all human chromosomes clearly showing that while some chromosomes (8,9,16) do show a great excess of maternal cDNMs, chromosome 17 (where the NF1 gene is located) presents almost only "paternal" cDNMs (figure 2a and comment in the last paragraph of page 488). Therefore, in our opinion the overall conclusions of the work from Goldmann et al. are not in contrast with our hypothesis, rather might reinforce it.

REFERENCES

1. Goldmann, J.M., Seplyarskiy, V.B., Wong, W.S.W., Vilboux, T., Neerincx, P.B., Bodian, D.L., Solomon, B.D., Veltman, J.A., Deeken, J.F., Gilissen, C., Niederhuber, J.E. (2018) Germline de novo mutation clusters arise during oocyte aging in genomic regions with high double-strand-break incidence. *Nat Genet*, **50**(4):487-492. doi: 10.1038/s41588-018-0071-6.

2. Garcia, B., Catasus, N., Ros, A., Rosas, I., Negro, A., Guerrero-Murillo, M., Valero, A.M., Duat-Rodriguez, A., Becerra, J.L., Bonache, S., Lázaro Garcia, C., Comas, C., Bielsa, I., Serra, E., Hernández-Chico, C., Martin, Y., Castellanos, E., Blanco, I. (2022) Neurofibromatosis type 1 families with first-degree relatives harbouring distinct NF1 pathogenic variants. Genetic counselling and familial diagnosis: what should be offered? *J Med Genet*, jmedgenet-2021-108301. doi: 10.1136/jmedgenet-2021-108301. Epub ahead of print.

3. Belyeu, J.R., Sasani, T.A., Pedersen, B.S., Quinlan, A.R. (2021) Unfazed: parent-of-origin detection for large and small de novo variants. *Bioinformatics*, **37**(24):4860–1. doi: 10.1093/bioinformatics/btab454.

SUPPLEMENTARY FIGURES AND LEGENDS

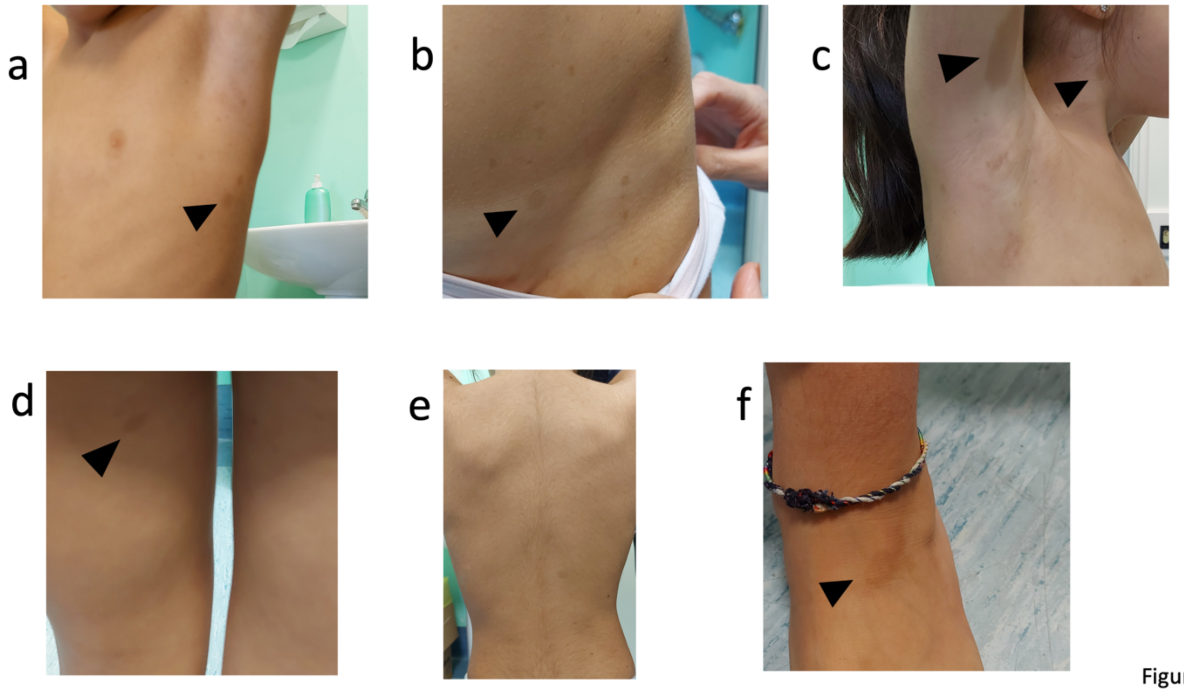

Figure S1

Supplementary Figure S1. Cutaneous clinical signs in case 2

Axillary freckling (**a**) and café-au-lait macules (CALs) (**b**); Inguinal freckling and CALs (**c**); sparse hairs in patient's 2 back (**d**) and feet (**e**). Black arrowheads point at larger CALs.

NF1 exon 21

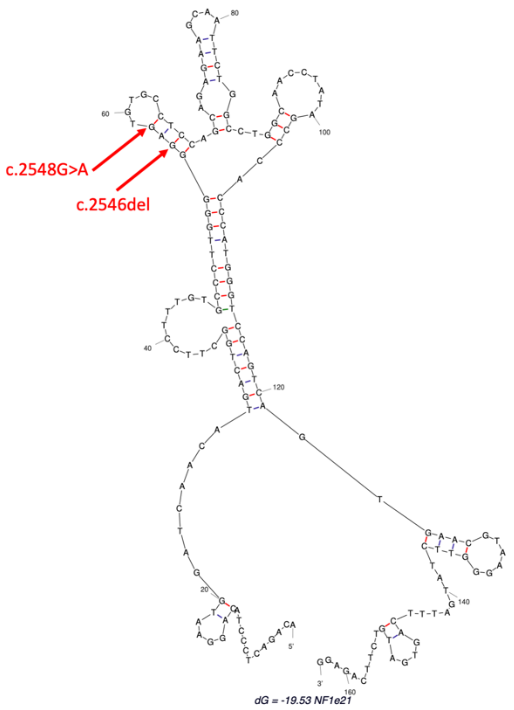

FIGURE S2

Supplementary Figure S2. Results of the UNAFold analysis for NF1 exon 21 double mutation

Secondary structure built by UNAFold for the region surrounding variants c.2546del and c.2548G>A (from nucleotide 2491 to 2653 of NF1 transcript NM_000267.3). Red arrows point at positions of variants.

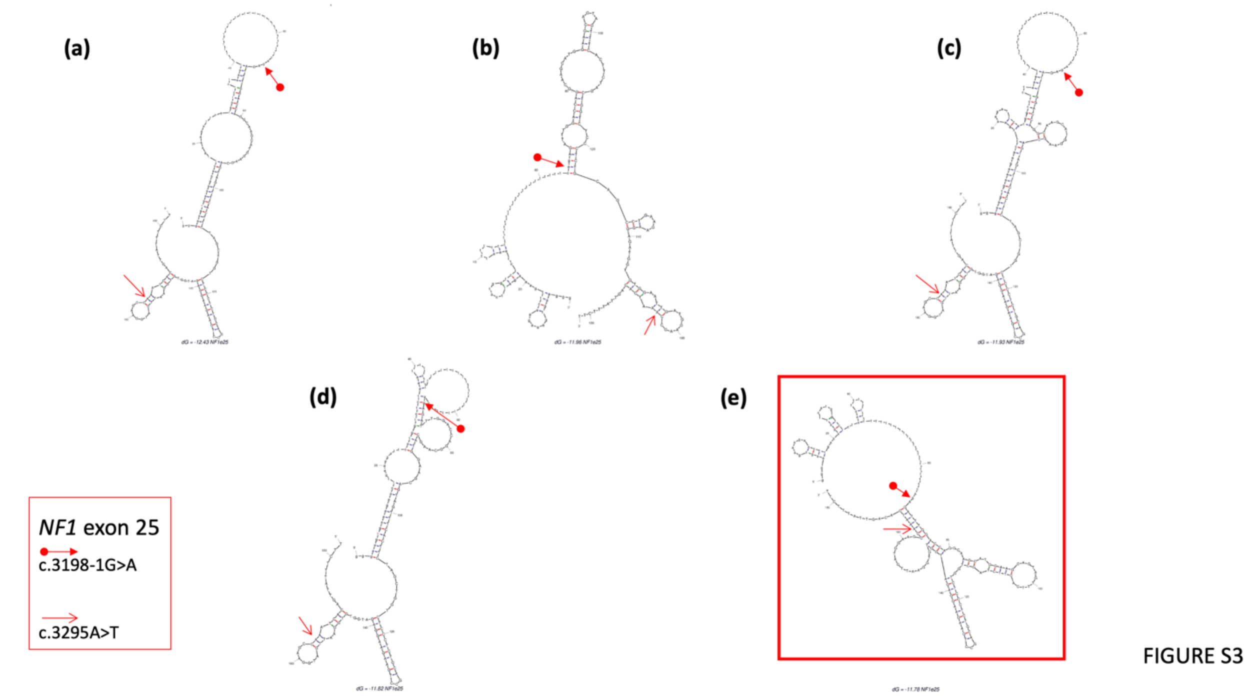

Supplementary Figure S3. Results of the UNAFold analysis for NF1 exon 25 double mutation

(**a-e**) Secondary structures built by UNAFold for the region surrounding mutations c.3198-1G>A and c.3295A>T (from nucleotide c.3198-67 to c.3313 of NF1 transcript NM_000267.3). A red box highlights the structure (**e**) where the two mutations come in close proximity. Red arrows point at positions of variants.

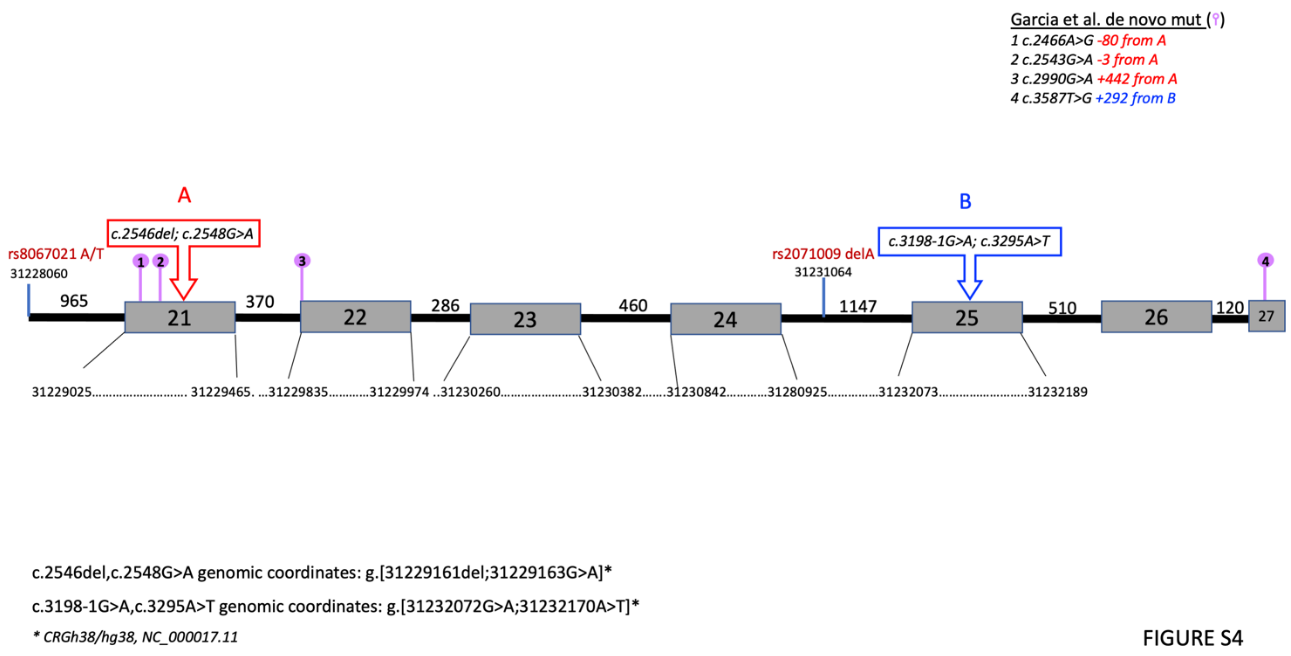

Supplementary Figure S4. Position of doublets g.[31229161del;31229163G>A] (A) and g.[31232072G>A;31232170A>T] (B) with respect to the four *de novo* variants reported by Garcia et al. (2022)

Position of the two doublets reported in this manuscript (boxed) with respect to the four *de novo* variants (purple lollipops) reported by Garcia et al. (2022). Introns are represented by a black solid line with size in nucleotides above. Grey boxes represent exons (drawn not to scale, genomic coordinates below). In red are indicated the SNPs (genomic coordinates below) used by us and by Garcia et al. (2022) to identify the parent of origin of the *de novo* variants. Top right, the distance of our doublets from the four de novo variants reported by Garcia et al. (2022) is indicated.
